# The Pattern of Mandibular Third Molar Impaction and Assessment of Surgery Difficulty: A Retrospective Study of Radiographs in East Baltic Population

**DOI:** 10.3390/ijerph18116016

**Published:** 2021-06-03

**Authors:** Aleksandra Jaroń, Grzegorz Trybek

**Affiliations:** Department of Oral Surgery, Pomeranian Medical University in Szczecin, Powstańców Wielkopolskich 72/18, 70-111 Szczecin, Poland; jaronola@gmail.com

**Keywords:** third molars, wisdom teeth, impaction, impacted teeth, impacted mandibular third molar, oral surgery, classifications of impaction, difficulty assessment

## Abstract

Classifications of impacted teeth allow defining the type and degree of retention, as well as assessing the degree of difficulty of the procedure. The aim of this study was to conduct retrospective analysis of the degree of retention and difficulty in the surgical removal of impacted mandibular third molars in the clinical material of the Department of Oral Surgery in 2013–2018. This study included 1585 dental panoramic radiographs of patients of the Department of Oral Surgery, who reported in 2013–2018, in order to perform surgical removal of the impacted mandibular third molar. Based on dental panoramic radiographs, the degree of retention was determined based on classifications according to Winter, according to Pell and Gregory, according to Tetsch and Wagner, and according to Asanami and Kasazaki. The difficulty of the procedure was also assessed based on the Pederson index. The most common types of lower wisdom tooth impaction are as follows: in Winter’s classification, mesial-angular impaction; in Tetsch and Wagner’s classification, oblique medial-angular impaction; in Pell and Gregory’s classification, impaction grade 2A; and in Asanami and Kasazaki’s classification, 3A and anterior inclination. In most cases of surgical removal of an impacted tooth, the anticipated difficulty of the procedure was rated as very difficult.

## 1. Introduction

Tooth impaction is one of the most common abnormalities of tooth position [1]. An impacted tooth (dens retens) is a tooth with a fully formed root, with complete development, which is partially or completely covered by hard and/or soft tissues, being outside the physiological period of eruption [2]. The procedure for surgical removal of impacted wisdom teeth is routinely performed in a dental surgery. Published studies have reported prevalences of impacted teeth that have ranged from 6.9 to 76.6% [3,4]. The most common of all teeth to be impacted are third molars, especially in the mandible [5,6]. They are among the most commonly impacted teeth next to the maxillary canine and mandibular second premolar [2].

The causes of third molar impaction can be divided into general and local [7,8]. In recent decades, an increase in the phenomenon of impaction has been noted. The explanation for this trend seems to be an increasing level of hygiene, as well as less frequent tooth loss and the influence of the lack of physiological tooth attrition due to changes in dietary habits [8]. The genetic etiology of third molar impaction plays an important role in the process of odontogenesis.

The MSX1 and AXIN2 genes may be considered as components of the genetic background, characterized by variable expressivity. As such, they promote tooth impaction in circumstances where specific environmental factors coexist and depending on the presence of other modulating genetic factors, additionally increasing the risk and stimulating the onset of abnormalities and/or increasing the degree of phenotypic manifestation of symptoms—such as the number of impacted teeth in the carrier [9]. Two-dimensional panoramic radiographs found that the initial mineralization time of mandibular third molar germs is 8–9 years old. The time of initial mineralization is individually variable [10]. The time of eruption of mandibular third molars is most often 17–20 years old [11]. The surgical removal of impacted wisdom teeth is a difficult procedure due to their different spatial locations and relationships with the surrounding anatomical structures. It is associated in most cases with the occurrence of perioperative complications, as well as with a significant reduction in the patient’s quality of life in the postoperative period [12]. Adequate assessment of the spatial location of the wisdom tooth and the difficulty of the procedure, as well as knowledge of the potential surgical complications associated with intervention into the maxillary bone tissues, is essential in planning the procedure.

The most commonly used method of radiographic examination in preoperative diagnosis to determine the position of the wisdom teeth is the panoramic radiograph [10,13]. X-ray diagnostics allows a proper diagnosis to be made, as well as establishing the methodology for clinical management. Due to the limitations of panoramic radiography, cone beam computed tomography is increasingly used [14].

Classifications of impacted third molars allow us to determine the degree of impaction and determine the best methodology for the surgical procedure. Planning the procedure based on the subject, physical examination, and additional investigations such as radiographs makes it possible to reduce the risk of complications. The authors of this publication use various classifications. Each of them is characterized by certain limitations. In the literature and clinical practice, authors usually use the following classifications: Winter, Tetsch and Wagner, Pell and Gregory, Asanami and Kasazaki [15,16,17,18].

There are many studies available in the literature presenting the problem of impacted mandibular third molars. The description of their impaction in most studies deals only with one classification, without considering the influence of the spatial position in relation to the occlusal plane line and in relation to the anterior margin of the mandibular ramus on the difficulty of the surgical procedure.

The inspiration to research the issues in this paper was the lack of studies regarding the assessment of the type of impaction and the degree of difficulty of the procedure discussing the problem among an East Baltic population. This topic is important due to the increasing prevalence of surgical removal of impacted third molars in the mandible. Classifications of the impacted teeth allow defining the type and degree of retention, as well as assessing the degree of difficulty of the procedure. Together with the operator’s experience, this affects the risk of complications associated with the procedure that may be minimized by targeted elective procedures of surgical removal of the impacted wisdom tooth. 

The aims of this study were as follows:

1. To assess the pattern of impacted mandibular third molars in an East Baltic population;
2. To assess the difficulty of surgical removal of impacted mandibular third molars in an East Baltic population.

## 2. Materials and Methods

### 2.1. Methodology of the Study

The analysis of radiographic images of impacted third molars in the mandible was based on pantographic radiographs taken digitally with a Cranex3Dx camera (Soredex, Tuusula, Finland) with a magnification ratio of 1:1.19. The analysis of digital pantographic images was performed using the Scanora 5.2.6 computer program (Soredex, Tuusula, Finland). The long axes of the second molar, the impacted third molar, the occlusal plane, the greatest width of the crown of the impacted molar, the tangent line to the anterior edge of the mandibular ramus, and the distal surface of the second lower molar were determined on the pantographic images. Based on the above data, the impaction degree of the impacted tooth was determined according to the classifications by Winter, Tetsch and Wagner, Pell and Gregory, and Asanami and Kasazaki, and the difficulty of the procedure was determined according to Pederson.

#### 2.1.1. Plotting the Lines Necessary to Evaluate the Impaction of an Impacted Mandibular Third Molar

The following lines were plotted to determine the impaction of the impacted third molar:

- Long axis of the tooth (a);
- Occlusal plane (A);
- Tangent to the anterior margin of the mandibular ramus (B).

**Figure 1:**
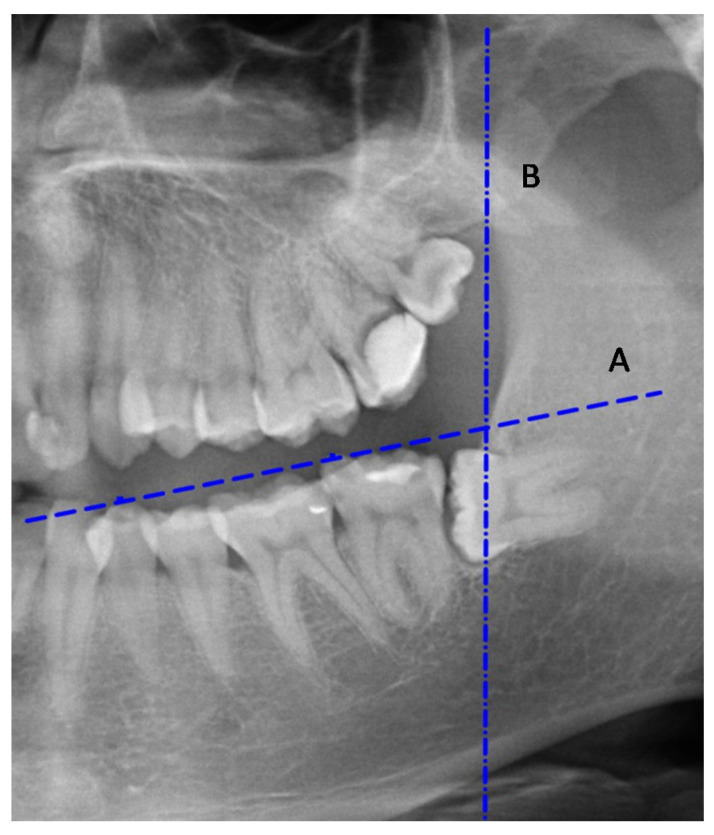
The occlusal plane line (**A**) and the mandibular anterior ramus line (**B**).

The occlusal plane line (A) is a straight line passing through the buccal cusp of the first mandibular premolar and the proximal buccal cusp of the second lower molar [13] (Figure 1).

The mandibular ramus anterior margin line (B) is a line tangent to, and parallel to, the greatest concavity of the mandibular ramus anterior margin [14] (Figure 1).

**Figure 2:**
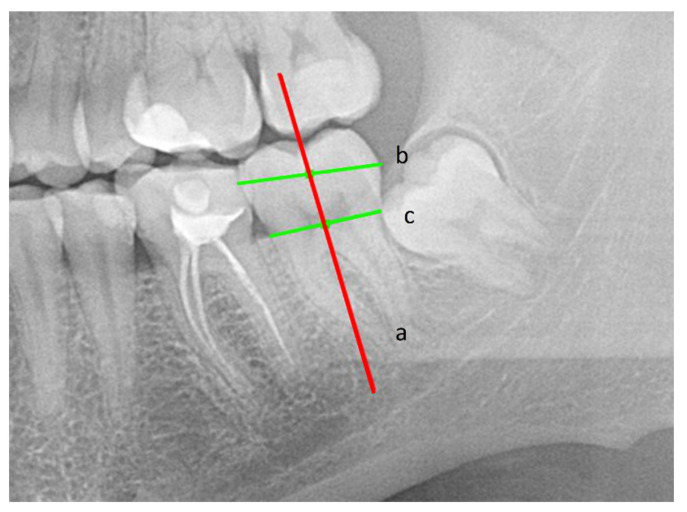
The long axis of the tooth (**a**), greatest width of the tooth crown (**b**), tooth neck (**c**).

To determine the long axis of the tooth (a), two points were determined: one at the midpoint of the greatest width of the tooth crown (b), and the other at the midpoint of the width of the tooth neck (c). A straight line drawn through the above points determined the long axis of the tooth [19] (Figure 2).

#### 2.1.2. Classification According to Winter

G. B. Winter documented impaction types based on angulation—the inclination of the crown of an impacted third molar—concerning the angle formed between the long axes of the second and third lower molars [15,20].

1. Vertical impaction—the long axes of the second molar and the impacted third molar are parallel;
2. Mesioangular impaction—the long axes of the second molar and the impacted third molar are coincident docoronally;
3. Distal-angular impaction—the long axes of the second molar and impacted third molar are convergent apically;
4. Horizontal impaction—the long axes of the second molar and impacted third molar are at right angles;
5. Buccolingual impaction—each tooth is oriented in a buccolingual direction;
6. Inverted impaction;
7. Other orientation.

#### 2.1.3. Classification by Pell and Gregory

This classification determines the degree of impaction of the third molar in the vertical and horizontal dimensions. It states the degree of impaction concerning the occlusal plane: A, B, C (vertical dimension), and the mandibular ramus: 1, 2, 3 (horizontal dimension) [16,21].

Position concerning the occlusal plane:

A: The occlusal surface of the third lower molar is either above or at the level of the occlusal plane;

B: The occlusal surface of the third lower molar is between the occlusal plane and the neck of the second molar;

C: The occlusal surface of the third lower molar is below the neck of the second molar.

Position concerning the anterior margin of the mandibular ramus:

Class 1: The distance between the distal surface of the second molar and the anterior margin of the mandibular ramus is greater than the anteroposterior dimension of the crown of the third lower molar;

Class 2: The distance between the distal surface of the second molar and the anterior margin of the mandibular ramus is less than the anteroposterior dimension of the crown of the third lower molar;

Class 3: The complete absence of space between the distal surface of the second molar and the anterior margin of the mandibular ramus.

#### 2.1.4. Classification According to Tetsch and Wagner

This classification determines the form of impaction of the third lower molar based on the angle of the long axis of this tooth to the occlusal plane [17].

1. Vertical impaction—the tooth is aligned parallel to the adjacent molars, at an angle of approximately 90° to the occlusal plane.
2. Horizontal impaction—the tooth is aligned parallel to the occlusal plane, at an angle of approximately 0°; depending on the direction of the position of the crown of the tooth, an additional distinction is made.
3. Sagittal impaction:
  - Medial-angular (the impacted third molar faces the occlusal surface to the second molar);
  - Distal-angular (the impacted third molar faces the occlusal surface to the anterior edge of the mandibular ramus).
4. Cross-impaction:
  - Buccal-angular (impacted third molar faces the occlusal surface to the buccal side);
  - Lingual-angular (impacted third molar faces the occlusal surface toward the lingual side).
5. Oblique impaction—the tooth is inclined concerning the occlusal plane in different variants, depending on the course of the long axis of the tooth concerning the occlusal plane from 0 degrees to 90 degrees.
  - Medial-angular;
  - Distal-angular;
  - Lingual-angular;
  - Bucco-angular;
6. Displacement impaction.

#### 2.1.5. Classification by Asanami and Kasazaki

This classification describes the degree of impaction of the third lower molar in both the vertical and horizontal dimensions concerning the angulation of this tooth. The degree of impaction of the tooth in the vertical dimension is determined by A, B, C, the horizontal dimension, and the inclination of the long axis of the third lower molar relative to the long axis of the second lower molar (1–3, 1–4) [18].

1. Vertical position:

A—Minor impaction;

B—Average impaction;

C—Deep impaction.

1—The distance between the distal surface of the second lower molar and the anterior margin of the mandibular ramus is greater than the horizontal dimension of the crown of the impacted tooth;

2—The distance between the distal surface of the second lower molar and the anterior edge of the mandibular ramus is equal to the horizontal dimension of the crown of the impacted tooth;

3—The distance between the distal surface of the second lower molar and the anterior edge of the mandibular ramus is less than the horizontal dimension of the crown of the impacted tooth.

2. Distal inclination:

A—Minor impaction;

B—Average impaction;

C—Deep impaction.

1—The distance between the distal surface of the second lower molar and the anterior margin of the mandibular ramus is greater than the horizontal dimension of the crown of the impacted tooth;

2—The distance between the distal surface of the second lower molar and the anterior edge of the mandibular ramus is equal to the horizontal dimension of the crown of the impacted tooth;

3—The distance between the distal surface of the second lower molar and the anterior edge of the mandibular ramus is less than the horizontal dimension of the crown of the impacted tooth.

3. Anterior inclination:

A—Minor impaction;

B—Average impaction;

C—Deep impaction.

1–4—The degree of inclination of the long axis of the impacted tooth of the third molar relative to the long axis of the second lower molar, depending on the value of the acute angle, is classified in degrees from 1 to 4. The authors Asanami and Kasazaki do not provide a reference value of the angle that determines the degree of inclination of the preceding in each point from 1 to 4.

4. Horizontal position:

A—Minor impaction;

B—Average impaction;

C—Deep impaction.

1—The distance between the distal surface of the second lower molar and the anterior margin of the mandibular ramus is greater than the horizontal dimension of the crown of the impacted tooth;

2—The distance between the distal surface of the second lower molar and the anterior edge of the mandibular ramus is equal to the horizontal dimension of the crown of the impacted tooth;

3—The distance between the distal surface of the second lower molar and the anterior edge of the mandibular ramus is less than the horizontal dimension of the crown of the impacted tooth.

5. Horizontal lingual position;
6. Lingual inclination;
7. Horizontal buccal position;
8. Buccal inclination;
9. Inverted position.

#### 2.1.6. Preoperative Assessment of the Difficulty of Surgical Removal of an Impacted Third Molar in the Mandible

The evaluation of the difficulty of surgical removal of an impacted third molar in the mandible was performed using the Pederson index [22,23]. Based on the impaction according to Winter, the spatial position of the impacted teeth was defined as mesial-angular alignment, horizontal/reversed alignment, vertical alignment, and distal-angular alignment. Concerning the occlusal plane (A, B, C) and the distance of the distal surface of the second molar from the tangent line to the mandibular ramus (1, 2, 3), the position of the impacted tooth was determined according to Pell and Gregory’s classification. Each parameter was assigned a score: mesioangular alignment—1 point, horizontal/reversed alignment—2 points, vertical alignment—3 points, distal-angular alignment—4 points, A—1 point, B—2 points, C—3 points, 1—1 point, 2—2 points, 3—3 points. The difficulty index was determined based on the sum of the scores obtained in the analysis of the pantographic examination. Accordingly, the degree of difficulty of surgical removal of the lower third molar was defined as slightly difficult for scores of 3–4 points, moderately difficult for 5–6 points, and very difficult for scores of 7–10 points.

### 2.2. Methodology of Statistical Analysis

Statistical analysis was performed using the statistical package R-version 3.5.2 [24]. Quantitative variables were described using standard measures of variability and location such as the arithmetic mean with standard deviation, quartiles: Q1, Q3, and median: Q2.

## 3. Results

### 3.1. Characteristics of the Study Group

We analyzed 1583 pantographic images of patients qualified for surgical removal of an impacted third molar in the mandible with local anesthesia (*n* = 1583). A total of 63.04% were female (*n* = 998), and 36.96% were male (*n* = 585). The mean age of the subjects was 26.95 years (±8.48), and the age groups were selected so that the number of subjects in each group was similar. A total of 50.16% (n = 794) were left and 49.84% (*n* = 789) were right lower wisdom teeth. Complete impaction was diagnosed in 56.79% (*n* = 899) of cases, asymptomatic partial impaction in 30.45% (n = 482), and symptomatic impaction in 12.76% (*n* = 202). In most of the surgical removal procedures of the impacted teeth, the anticipated difficulty of the procedure was rated as very difficult (39.54%). Detailed results are shown in Table 1 and Table 2.

### 3.2. Winter Characteristics of Lower Wisdom Teeth Position

In Winter’s impaction classification, the most numerous group was third wisdom teeth in the mesial-angular position (*n* = 832), and a slightly less numerous group was third molars in the distal-angular position (*n* = 618). Inverted impaction (*n* = 7) and impaction described as other (*n* = 1) were the least frequent. A detailed summary of the degrees of impaction according to Winter is shown in Table 3.

### 3.3. Position Characteristics of Lower Wisdom Teeth According to Pell and Gregory

The most common position of impacted mandibular third molars according to the amount of space between the anterior margin of the mandibular ramus and the second lower molar (1, 2, 3) was distance 2 (70.44%), and the least common was position 3 (10.68%). In the evaluation of the impaction depth (A, B, C), as many as 50.6% of cases showed depth A and only 9.67% showed depth C. Grade 2A was the most abundant impaction grade according to Pell and Gregory (36.26%), with 1C (2.21%) and 3C (1.83%) being the least abundant. A detailed comparison of impaction grades in the study group is shown in Table 4.

### 3.4. Position Characteristics of Lower Wisdom Teeth According to Tetsch and Wagner

In most cases, impacted wisdom teeth occurred in the oblique medial-angular position (*n* = 833). Inverted impaction (0.44%) and impaction described as other (0.06%) were observed the least. The results of the analysis of the number of different degrees of impaction according to Tetsch and Wagner are shown in Table 5.

### 3.5. Position Characteristics of Lower Wisdom Teeth According to Asanami and Kasazaki

In this classification, anterior inclination was observed most frequently (52.56%), and inverted (0.44%) and impaction described as “other” (0.06%) were observed least frequently. Depth (A, B, C) was found most frequently with A (50.6%), and depth was found least frequently with C (9.67%). The distance of the anterior edge of the mandibular ramus from the distal surface of the second lower molar (1, 2, 3) was evaluated for all impaction types except for anterior inclination, inverted position, and other impaction (*n* = 840). Among the other impaction types, the most common distance was 3 (39.86%). The position characteristics of lower wisdom teeth are shown in Table 6.

## 4. Discussion

### 4.1. Position Characteristics of Mandibular Impacted Third Molar

Classifications of the spatial location of impacted lower third molars allow us to determine the degree of impaction of the tooth, which allows preoperative determination of the degree of difficulty of the procedure and the best methodology for the surgical removal procedure [25]. In the literature, authors most commonly use the classifications of Winter, Tetsch and Wagner, Pell and Gregory, and Asanami and Kasazaki.

Winter’s classification is the most commonly chosen method for spatial assessment of impacted teeth in the literature because of its simplicity of use. It does not require the use of additional measuring instruments, which influences its widespread use in clinical practice.

Our study showed that the most common type of impaction, at 52.56%, according to Winter, is mesial-angle impaction. Padhye et al. presented an analysis of pantographic radiography of 1200 subjects, in which 33.33% of the subjects showed mesial-angle alignment according to Winter [26]. Kumar et al. observed the prevalence of mesial-angle alignment in 52.89% of cases in Eritrean residents [27]. These reports were confirmed in 2016 by Nagaraj and co-authors [28] presenting mesial-angle impaction in 47.1% of patients, as well as many other researchers [29,30,31,32]. In the studies of Al-Dajani et al. [33] and Yilmaz et al. [34], vertical impaction was found to be the most common position. The first team showed the occurrence of this impaction in 40.7% and mesioangular impaction in only 7.1% of patients; the second team showed vertical impaction in 53% and mesioangular impaction in 29% of patients. The differences in results may be due to the adoption of an incorrect modification of Winter’s index in the studies of Al-Dajani et al. [33] and Yilmaz et al. [34]. The researchers determined the long axes of the second and third molars to determine the angulation of the impacted molar. The reference point in the measurements was the axis of the second tooth. The angle of deviation of the third tooth axis from the second tooth axis was measured. If the deviation from the reference line was 10° to either side, tooth impaction was defined as vertical. Winter’s [15] original classification, by design, does not give a value for the angle of tooth deviation and the possibility of deviation from the long axis of the second tooth, but only the spatial relationship of the long axes. It may seem that this modification is identical to the angles adopted in the Tetsch and Wagner classification; however, Al-Dajani and Yilmaz measured the amplitude from the axis of the second tooth, contrary to the Tetsch and Wagner classification, which measures the angle between the occlusal plane and the axis of the impacted tooth. Researchers from Honk Kong [4] presented horizontal impaction as the most common type. In their study of 7486 patients, with 42.45% having impacted lower wisdom teeth, they showed the presence of horizontal impaction in 47.45% of the patients analyzed. In our study, the second most common impaction (39.04%) was distal-angular alignment. The results of this study confirm the findings of Goyal et al. [35], who presented the same order of prevalence of each impaction type according to Winter. In a sample size of 700 subjects, of which 40.7% were impacted mandibular third molars, 53.89% of the teeth were in the mesial-angular position, and 20.56% were in the distal-angular position. The same pattern of prevalence of individual impactions was presented by Al-Anqudi et al. [30]. The predominant second most common impaction in the literature is the vertical position [36,37]. The variability in results regarding the incidence of vertical and distal-angular impaction may be related to the often slight degree of deviation of the crown of the impacted tooth from the long axis of the second molar, which is misinterpreted by the authors of the publications as vertical alignment. In our study, impaction was assumed to be defined as vertical only when the long axes of the mandibular second and third molars were parallel, with no possible deviation of their axes. In the literature, authors of publications unanimously present the occurrence of inverted or other impaction as the least frequent, which was confirmed in our study [4,29,30,31,32,36,37].

Analogous to Winter’s classification, impacted third molars in the mandible are graded based on molar angulation according to Tetsch and Wagner. Unfortunately, one publication was found in which the above classification was present. A Polish team [10] presented an evaluation of the structure and impaction type of impacted third molars based on the above classification. The study evaluated 100 pantographic radiographs of patients of the Silesian Medical University in Poland considering both upper and lower wisdom teeth. The researchers presented only teeth with vertical impaction, mesial-angular impaction, distal-angular impaction, and displacement. The frequency of impaction degrees was 16%, 40%, 13%, and 1%, respectively. Many authors incorrectly use a pie chart based on the Tetsch and Wagner classification in assessing the degree of impaction according to Winter [34,38].

Winter’s and Tetsch and Wagner’s classifications consider only the angulation of the impacted molar. They do not take into account the amount of space in the arch available for the eruption of the wisdom tooth. Pell and Gregory determined the degree of impaction based on two variables—the depth of impaction of the tooth and the space available between the anterior margin of the mandibular ramus and the distal surface of the lower second molar. Assessment of the spatial location according to Pell and Gregory is often presented by authors of previous publications [27,31,34,37,39,40,41]. Abbas and co-authors presented a study on 358 impacted molars in the mandible. They evaluated the spatial position according to Pell and Gregory, and the occurrence of pathologies associated with the impacted tooth. Position A impaction depth was the most common with 43.02%. A total of 65.95% of the teeth were classified as impaction 2 according to Pell and Gregory. The researchers also determined molar angulation; however, they did not provide the methodology of the study or the classification by which it was assessed. Eshghpour et al. evaluated the impaction rate of impacted mandibular third molars in a northern Iranian population [31]. Based on 1397 cases of impacted mandibular third molars, they identified the most common types of impaction. Concerning the occlusal plane, the most common impaction type was B (*n* = 892; 64.85%). Concerning the mandibular ramus, 2 was the most common (*n* = 677; 48.46%). Researchers from Pakistan determined the degree of impaction of 100 wisdom teeth in the mandible during a year-long study [37]. The authors used letter and numerical terms interchangeably, which may confuse interpreting their results. They used the letter designation to refer to the position of the tooth relative to the mandibular ramus, and the number designation to refer to the depth of impaction. The most common type of impaction relative to the mandibular ramus was impaction A with 39% (impaction 1 in the original Pell and Gregory classification and in our study); however, the incidence of impaction B (impaction 2 in the Pell and Gregory classification and in our study) was slightly lower with 35%. Concerning the occlusal plane, the most common impaction was class 2—45% (impaction B in the original classification by Pell and Gregory and in our study); however, the difference between classes 2 and 1 was small (42%). The rarest was class 3—13% (impaction C in the original classification according to Pell and Gregory and in our study). The same order of frequency of impaction groups was presented by Prajapati and co-authors [41]. They conducted a study to evaluate the spatial position of impacted third molars in the mandible using Winter’s and Pell and Gregory’s classifications and also the relationship of the occurrence of caries of the second molar adjacent to the impacted tooth. The authors observed the presence of class 1 in the majority of cases (62.60%). The researchers did not observe the occurrence of class 3 impaction, which may be related to the small group (*n* = 200). The most common depth of impaction in the subjects was B impaction (47.82%). Obiechina et al. in a study based on 473 impacted mandibular third molars presented 54.55% of cases in the A position, 31.92% in the B position, and 13.53% in the C position. Regarding the mandibular ramus, 22.62% of the teeth were in position 1, 60.89% in position 2, and 16.49% in position 3. The study was limited to patients aged 16–45 years [39].

The authors divided the impacted teeth into prophylactic and symptomatic teeth. The only parameter for inclusion in the symptomatic teeth group was the presence of pain. As many as 68.29% of the cases were symptomatic teeth. The connection between the degree of impaction and the occurrence of pain was also evaluated. Different results were reported in a study on 732 impacted lower wisdom teeth by Yilmaz et al. [34]. In up to 61%, the impaction depth, according to Pell and Gregory, was level C. The researchers did not provide a class breakdown of the relationship of the tooth position to the mandibular ramus; they only calculated the size of the sinus space in millimeters. The alveolar space is the distance of the anterior edge of the mandibular ramus from the distal surface of the second molar. Unfortunately, this magnitude has not been related to the anteroposterior dimension of the crown of the impacted tooth. An analysis of the current scientific literature leads to the conclusion that the preponderance of research teams only considers the frequency of occurrence of each degree of impaction depth and reference to the anterior edge of the mandibular ramus, without considering the relationship between depth and distance indices. Kumar and co-authors conducted a retrospective study on an Eritrean population (*n* = 552) in which they evaluated the impaction degree of impacted mandibular third molars based on Winter’s and Pell and Gregory’s classifications [27]. The most common classification was 1A (59.78%), the least common classifications were 3A (0.36%) and 3B (0.36%), and classification 3C did not occur. Similarly, impaction grades according to Pell and Gregory were classified by researchers from Iran [20]. In their study, they evaluated 1165 impacted third molars, of which 64.4% were teeth in the mandible. The most common impaction types in the study were 2A (38.93%) and 2B (17.67%), and the least common was 3A (1.87%).

In our study, the most common grade of impaction depth was grade A (50.60%). Concerning the anterior margin of the mandibular ramus, the impacted molar was most common in position 2 (70.44%). Additionally, the association between depth and distance was also considered. The most common impaction grades were 2A (36.26%) and 2B (28.55%). Type 3C was the least common (1.83%).

Due to the limitations of the classifications described above, Asanami and Kasazaki in 1990 created an impaction division that takes into account both angulation and the spatial position of the molar relative to the occlusal plane and concerning the anterior margin of the mandibular ramus. Unfortunately, Asanami and Kasazaki’s classification is used very rarely in the literature. Only one publication on antibiotic therapy in the prevention of postoperative complications in third molar surgery by a research team from Krakow [42] classified lower third molars according to Asanami and Kasazaki.

In our study, the results of the classification of impacted wisdom teeth in the mandible using the Asanami and Kasazaki divisions were presented. The most common grades of impaction were grade 3 distance (84.92%), depth A (50.60%), and anterior inclination (52.56%).

Despite the analogy of classification according to Asanami and Kasazaki and according to Pell and Gregory, the frequency of each impaction distance class was different. This is because the interpretation of numerical designations 1–3 according to Asanami and Kasazaki is different from Pell and Gregory. The frequency of individual distances also varies because the above classification does not evaluate distances for teeth in anterior inclination.

### 4.2. Evaluation of the Predicted Degree of Difficulty in the Surgical Removal of an Impacted Mandibular Third Molar

The degree of difficulty according to Pederson takes into account both the angulation of the molar and its spatial position relative to the mandibular ramus and the occlusal plane. The analysis of impaction degrees in the above-mentioned classifications already allows a preliminary assessment of the difficulty of the procedure. The numerical value of the Pederson index is the highest for the distal-angle position according to Winter (4 points), depth C (3 points), and distance 3 (3 points). With the presence of only one of the parameters in the highest value, extraction will be at least moderately difficult. In our study, we were able to draw preliminary conclusions already from the analysis of impaction degrees that extraction of third molars is more difficult in women. They had significantly more distal-angular impactions than men. It was confirmed by statistical analysis that the procedures were significantly more difficult in women than in men.

Park [43] evaluated the difficulty of surgical removal of third lower molars using the Pederson scale and the subjective feeling of the operator. They evaluated 762 impacted teeth scheduled for surgical removal between 2009 and 2014. Molar angulation according to Winter and impaction type according to Pell and Gregory were assessed, and the results were converted to a numerical score on the Pederson scale. A total of 45.53% of women participated in the study. The procedures were more difficult in men. There was no significant difference in the predicted difficulty of the procedure between the sexes. The authors of the study noted the differences in procedure difficulty as assessed by the operator and the Pederson index value. The study was performed on a group almost 2.5 times smaller than in our study, which may have influenced the different results. According to the author of the study, the most difficult procedures were in the group over 50 years old. The treatments were easiest in the youngest patients, between 10 and 19 years of age [43]. In the literature, authors have reported a relationship between the position of the impacted mandibular third molar and mandibular fractures. Kumar et al. demonstrated in a study of 64 subjects that a deeper position of the impacted tooth led to an increased risk of angle fracture and decreased risk of condylar fracture [44]. Constantinides et al. conducted a study on the effect of the type of anesthesia on postoperative complications concerning IAN. Nowadays, most mandibular third molar removal procedures are performed under local anesthesia. Only atypical position, extreme difficulty of the procedure, location in spaces that are difficult to access intraorally, and mental illness or anxiety are indications to perform the procedure under general anesthesia. In our study, all patients met the criteria for having the surgical procedure under local anesthesia [45].

This study evaluated the predicted difficulty of surgical removal of impacted third molars in the mandible by age group. It was shown that the procedures were significantly easier in subjects under 20 years of age than in others (*p* < 0.001). Our study shows that the degree of difficulty increases with age until the age of 30 years (46.52% of very difficult procedures in the group of 26–30 years) and then slightly decreases in patients over 30 years of age (43.14%). It should be noted that statistically, the difficulty of the procedure decreases after the age of 30, but this is only based on the spatial location of the impacted tooth. Other factors such as the density of the bone matrix around the impacted third molar, its degree of ankylosis, and the patient’s weight also influence the clinical judgment, which is based on the operator’s experience [46,47,48]. This study has some limitations. Assessment of the position of the tooth based on a 2D panoramic image is not as accurate as that based on a 3D examination. In the next stages of this study, we plan to determine the dependence of gender and age on the position of the impacted tooth as well as comparing the position on 2D and 3D images. 

The development of 3D diagnostics, 3D printing, and computer methods may allow us to create computer programs that, after marking the measurement points, will determine the type of retention. Additionally, planning based on 3D imaging may allow creating surgical templates to help and allow for precise planning of the surgical procedure.

## 5. Conclusions

The most common types of lower wisdom tooth impaction are as follows: in Winter’s classification, mesial-angular impaction; in Tetsch and Wagner’s classification, oblique medial-angular impaction; in Pell and Gregory’s classification, distance from the anterior edge of the mandibular ramus 2, impaction depth A, and impaction grade 2A; and in Asanami and Kasazaki’s classification, distance of the mandibular ramus from the distal surface of the second lower molar 3, impaction depth A, and anterior inclination. In most cases of surgical removal of an impacted tooth, the anticipated difficulty of the procedure was rated as very difficult.

## Figures and Tables

**Table 1:** Characteristics of the study group.

		*n* (%)
Age	Average ± SD	26.95 ± 8.48
	Median	25
	Quartile	21–31
	Under 20	337 (21.29%)
	20–25	471 (29.75%)
	26–30	374 (23.63%)
	Over 3	401 (25.33%)
Sex	Female	998 (63.04%)
	Male	585 (36.96%)

SD—standard deviation. *n*—number of patients.

**Table 2:** Characteristics of impacted third molars in the mandible.

		*n* (%)
Tooth number	Tooth 38	794 (50.16%)
	Tooth 48	789 (49.84%)
Diagnosis	Retentio totalis	899 (56.79%)
	Retentio partialis asymtomathic	482 (30.45%)
	Retentio partialis symtomathic	202 (12.76%)
Difficulty of the procedure	Very difficult	626 (39.54%)
	Moderately difficult	596 (37.65%)
	Slightly difficult	361 (22.80%)

*n*—number of patients.

**Table 3:** Frequency of impaction degrees among impacted mandibular third molars according to Winter in the study group.

		*n* (%)
Classification according to Winter	Mesioangular	832 (52.56%)
	Distal-angular	618 (39.04%)
	Vertical	85 (5.37%)
	Horizontal	31 (1.96%)
	Buccolingual	9 (0.57%)
	Inverted	7 (0.44%)
	Other	1 (0.06%)

*n*—number of patients.

**Table 4:** Frequency of impaction degrees among impacted mandibular third molars according to Pell and Gregory in the study group.

		*n* (%)
Position concerning the anterior margin of the mandibular ramus	1	299 (18.89%)
	2	1115 (70.44%)
	3	169 (10.68%)
Position concerning the occlusal plane	A	801 (50.60%)
	B	629 (39.73%)
	C	153 (9.67%)
Classification by Pell and Gregory	1A	159 (10.04%)
	1B	105 (6.63%)
	1C	35 (2.21%)
	2A	574 (36.26%)
	2B	452 (28.55%)
	2C	89 (5.62%)
	3A	68 (4.30%)
	3B	72 (4.55%)
	3C	29 (1.83%)

*n*—number of patients.

**Table 5:** Frequency of impaction degrees among impacted mandibular third molars according to Tetsch and Wagner in the study group.

		*n* (%)
Classification according to Tetsch and Wagner	Oblique medial-angular	833 (52.62%)
	Oblique distal-angular	617 (38.98%)
	Vertical	85 (5.37%)
	Horizontal	20 (1.26%)
	Horizontal medial-angular	11 (0.69%)
	Buccolingual	9 (0.57%)
	Inverted	7 (0.44%)
	Other	1 (0.06%)

*n*—number of patients.

**Table 6:** Frequency of impaction degrees among impacted mandibular third molars according to Asanami and Kasazaki in the study group.

		*n* (%)
Horizontal position	1	51 (3.22%)
	2	61 (3.85%)
	3	631 (39.86%)
	Inability to assess	840 (53.06%)
Vertical position	A	801 (50.60%)
	B	629 (39.73%)
	C	153 (9.67%)
Classification by Asanami and Kasazaki	Anterior inclination	832 (52.56%)
	Distal inclination	574 (36.26%)
	Vertical	85 (5.37%)
	Distal position	44 (2.78%)
	Horizontal	31 (1.96%)
	Horizontal buccolingual	9 (0.57%)
	Inverted	7 (0.44%)
	Other	1 (0.06%)

*n*—number of patients.

## Data Availability

Data are available on request: kzchstom@pum.edu.pl.

## References

[1] Dimitroulis G. (1996). A Synopsis of Minor Oral Surgery.

[2] Peterson L.J., Ellis E.I.I.I., Hupp J.R., Tuker M.R. (1998). Principles of Management of Impacted Teeth. Contemporary Oral and Maxillofacial Surgery.

[3] Peltola J.S. (1993). A panoramatomographic study of the teeth and jaws of Finnish university students. Community Dent. Oral Epidemiol..

[4] Chu F., Li T., Lui V., Newsome P., Chow R., Cheung L. (2003). Prevalence of impacted teeth and associated pathologies—A radiographic study of the Hong Kong Chinese population. Hong Kong Med. J..

[5] Rahman A. (1994). Radiological Assessment of Surgery Difficulty of Impacted Mandibular Third Molar. Int. Med. J..

[6] Abu-Hussein M., Watted N. (2016). Prevalence of Impacted Mandibular Third Molars in Population of Arab Israeli: A Retrospective Study. IOSR-JDMS.

[7] Blondeau F., Daniel N.G. (2007). Extraction of impacted mandibular third molars: Postoperative complications and their risk factors. J. Can. Dent. Assoc..

[8] Szubert P., Jankowski M., Krajecki M., Jankowska-Wika A., Sokalski J. (2015). Analiza czynników predysponujących do powikłań po chirurgicznym usunięciu zębów mądrości w żuchwie. Dental Forum.

[9] Trybek G., Jaroń A., Grzywacz A. (2021). Association of Polymorphic and Haplotype Variants of the MSX1 Gene and the Impacted Teeth Phenomenon. Genes.

[10] Zawilska A., Koszowski R., Waśkowska J. (2007). Ocena budowy oraz typów retencji zatrzymanych trzecich trzonowców w obrazie pantomograficznym. Ann. Acad. Med. Stetin..

[11] Avery J. (2011). Oral Development and Histology.

[12] Sigron G.R., Pourmand P.P., Mache B., Stadlinger B., Locher M.C. (2014). The most common complications after wisdom-tooth removal: Part 1: A retrospective study of 1199 cases in the mandible. Swiss Dent. J..

[13] Młynarska-Zduniak E., Żyszko A. (1996). Położenie trzecich zębów trzonowych na zdjęciach pantomograficznych u osób z normą zgryzową. Czas. Stomatol..

[14] Matzen L.H., Wenzel A. (2014). Efficacy of CBCT for assessment of impacted mandibular third molars: A review—Based on a hierarchical model of evidence. Dentomaxillofa. Radiol..

[15] Winter G.B. (1926). Impacted Mandibular Third Molars.

[16] Pell G.J., Gregory G.T. (1933). Impacted mandibular third molars: Classification and modified technique for removal. Dent. Dig..

[17] Tetsch P., Wagner W. (1985). Operative Extraction of Wisdom Teeth.

[18] Asanami S., Kasazaki Y. (1990). Expert Third Molar Extractions.

[19] Ratajek-Gruda M., Grzesiak-Janas G., Białkowska-Głowacka J. (2004). Wyznaczanie osi długiej trzecich dolnych zębów trzonowych na zdjęciach pantomograficznych. Mag. Stom..

[20] Hashemipour M.A., Tahmasbi-Arashlow M., Fahimi-Hanzaei F. (2013). Incidence of impacted mandibular and maxillary third molars: A radiographic study in a Southeast Iran population. Med. Oral Patol. Oral Cir. Bucal..

[21] Vilela M.E., Amorim P. (2011). Study of position and eruption of lower third molars in adolescents. RSBO.

[22] Diniz-Freitas M., Lago-Mendez L., Gude-Sampedro F., SomozaMartin J.M., Gándara-Rey J.M., García-García A. (2007). Pederson scale fails to predict how difficult it will be to extract lower third molars. Br. J. Oral Maxillofac. Surg..

[23] Kharma M.Y., Sakka S., Aws G., Tarakji B., Nassani M.Z. (2014). Reliability of Pederson scale in surgical extraction of impacted lower third molars: Proposal of new scale. J. Oral Dis..

[24] R Core Team R: A Language and Environment for Statistical Computing.

[25] Gbotolorun O.M., Arotiba G.T., Ladeinde A.L. (2007). Assessment of factors associated with surgical difficulty in impacted mandibular third molar extraction. J. Oral Maxillofac. Surg..

[26] Padhye M.N., Dabir A.V., Girotra C.S., Pandhi V.H. (2013). Pattern of mandibular third molar impaction in the Indian population: A retrospective clinico-radiographic survey. Oral Surg. Oral Med. Oral Pathol. Oral Radiol..

[27] Kumar V.R., Yadav P., Kahsu E., Girkar F., Chakraborty R. (2017). Prevalence and Pattern of Mandibular Third Molar Impaction in Eritrean Population: A Retrospective Study. J. Contemp. Dent. Pract..

[28] Nagaraj T., Balraj L., Irugu K., Rajashekarmurthy S., Sreelakshmi (2016). Radiographic assessment of distribution of mandibular third molar impaction: A retrospective study. J. Indian. Acad. Oral Med. Radiol..

[29] Quek S.L., Tay C.K., Tay K.H., Toh S.L., Lim K.C. (2003). Pattern of third molar impaction in a Singapore Chinese population: A retrospective radiographic survey. Int. J. Oral Maxillofac. Surg..

[30] Al-Anqudi S.M., Al-Sudairy S., Al-Hosni A., Al-Maniri A. (2014). Prevalence and Pattern of Third Molar Impaction: A retrospective study of radiographs in Oman. Sultan. Qaboos. Univ. Med. J..

[31] Eshghpour M., Nezadi A., Moradi A., Shamsabadi R.M., Rezaei N.M., Nejat A. (2014). Pattern of mandibular third molar impaction: A cross-sectional study in northeast of Iran. Niger J. Clin. Pract..

[32] Elkhateeb S.M., Awad S.S. (2018). Accuracy of panoramic radiographic predictor signs in the assessment of proximity of impacted third molars with the mandibular canal. J. Taibah Univ. Med Sci..

[33] Al-Dajani M., Abouonq A.O., Almohammadi T., Alruwaili M.K., Alswilem R.O., Ibrahim A.A., Alzoubi A. (2017). Cohort Study of the Patterns of Third Molar Impaction in Panoramic Radiographs in Saudi Population. Open Dent. J..

[34] Yilmaz S., Adisen M.Z., Misirlioglu M., Yorubulutb S. (2016). Assessment of Third Molar Impaction Pattern and Associated Clinical Symptoms in a Central Anatolian Turkish Population. Med. Princ. Pract..

[35] Goyal S., Verma P., Raj S.S. (2016). Radiographic Evaluation of the Status of Third Molars in Sriganganagar Population—A Digital Panoramic Study. MJMS.

[36] Obuekwe O.N., Enabulele J.E. (2017). Gender Variation in Pattern of Mandibular Third Molar Impaction. Oral Disord. Ther..

[37] Wazir S., Khan M., Ashfaq M., Manzoor S. (2017). Evaluation of Third Molar Impaction Distribution and Patterns in a Sample of Lebanese Population. Pak. Oral Dental, J..

[38] Latt M.M., Chewpreecha P., Wongsirichat N. (2015). Prediction of difficulty in impacted lower third molars extraction; review of literature. M Dent. J..

[39] Obiechina A.E., Arotiba J.T., Fasola A.O. (2001). Third molar impaction: Evaluation of the symptoms and pattern of impaction of mandibular third molar teeth in Nigerians. Odontostomatol. Trop..

[40] Abbas G.A., Uraibi A.H. (2016). Assessment of impaction pattern and associated symptoms for mandibular third molar. Med. Princ Pract..

[41] Prajapati V.K., Mitra R., Vinayak K.M. (2017). Pattern of mandibular third molar impaction and its association to caries in mandibular second molar: A clinical variant. Dent. Res. J..

[42] Kaczmarzyk T., Wichlinski J., Stypulkowska J., Zaleska M., Panas M., Woron J. (2007). Single-dose and multi-dose clindamycin therapy fails to demonstrate efficacy in preventing infectious and inflammatory complications in third molar surgery. Int. J. Oral Maxillofac. Surg..

[43] Park K.L. (2016). Which factors are associated with difficult surgical extraction of impacted lower third molars?. J. Korean Assoc. Oral Maxillofac. Surg..

[44] Kumar S., Sinha R., Uppada U.K., Ramakrishna Reddy B.V., Paul D. (2015). Mandibular Third Molar Position Influencing the Condylar and Angular Fracture Patterns. J. Maxillofac. Oral Surg..

[45] Costantinides F., Biasotto M., Maglione M., Di Lenarda R. (2016). Local vs general anaesthesia in the development of neurosensory disturbances after mandibular third molars extraction: A retrospective study of 534 cases. Med. Oral Patol. Oral Cir. Bucal..

[46] Renton T., Smeeton N., Mcgurk M. (2001). Factors predictive of difficulty of mandibular third molar surgery. Br. Dent. J..

[47] Susarla S.M., Dodson T.B. (2005). How well do clinicians estimate third molar extraction difficulty?. J. Oral Maxillofac. Surg..

[48] Akadiri O., Obiechina A. (2009). Assessment of difficulty in third molar surgery—A systematic review. J. Oral Maxillofac. Surg..

